# Effects of cattle grazing on small mammal communities in the Hulunber meadow steppe

**DOI:** 10.7717/peerj.2349

**Published:** 2016-08-23

**Authors:** Chan Cao, Ling-Ying Shuai, Xiao-Ping Xin, Zhi-Tao Liu, Yan-Ling Song, Zhi-Gao Zeng

**Affiliations:** 1 Key Laboratory of Animal Ecology and Conservation Biology, Institute of Zoology, Chinese Academy of Sciences, Beijing, China; 2 University of Chinese Academy of Sciences, Beijing, China; 3 Institute of Agricultural Resources and Regional Planning, Chinese Academy of Agricultural Sciences, Beijing, China; 4 College of Life Science and Technology, Harbin Normal University, Harbin, China

**Keywords:** Hulunber, Grazing, Small mammals, Meadow steppe, Diversity, Intermediate disturbance hypothesis

## Abstract

Small mammals play important roles in many ecosystems, and understanding their response to disturbances such as cattle grazing is fundamental for developing sustainable land use strategies. However, how small mammals respond to cattle grazing remains controversial. A potential cause is that most of previous studies adopt rather simple experimental designs based solely on the presence/absence of grazing, and are thus unable to detect any complex relationships between diversity and grazing intensity. In this study, we conducted manipulated experiments in the Hulunber meadow steppe to survey small mammal community structures under four levels of grazing intensities. We found dramatic changes in species composition in native small mammal communities when grazing intensity reached intermediate levels (0.46 animal unit/ha). As grazing intensity increased, *Spermophilus dauricus* gradually became the single dominant species. Species richness and diversity of small mammals in ungrazed and lightly grazed (0.23 animal unit/ha) area were much higher than in intermediately and heavily grazed area. We did not detect a humped relationship between small mammal diversity and disturbance levels predicted by the intermediate disturbance hypothesis (IDH). Our study highlighted the necessity of conducting manipulated experiments under multiple grazing intensities.

## Introduction

As one of the most widespread forms of land use (Díaz et al., 2007), cattle grazing can shape the structure and functioning of grassland systems in many ways. Generally, grazing can remove plant biomass and decrease canopy height or coverage (e.g. Hickman & Hartnett, 2002; Rickart, Bienek & Rowe, 2013). Selective feeding and trampling of cattle may change relative abundance of different plant species, and alter composition and diversity of local plant communities (Augustine & McNaughton, 1998; Olff & Ritchie, 1998; Ausden et al., 2005). Cattle grazing and trampling may also affect some ecologically important traits of substrate such as soil hardness (Greenwood & McKenzie, 2001; Steffens et al., 2008) and nitrous oxide fluxes (Yan et al., 2016).

These habitat modifications caused by cattle can affect other sympatric organisms such as small mammals. Although, small in body size, small mammals are usually abundant in number and engaged in many important ecological processes such as seed dispersal (Li & Zhang, 2003), nitrogen cycling (Bakker et al., 2004) and carbon exchange (Liu et al., 2013). Small mammals feed on plants, fungi and insects, while provide food for many predators (Verts & Carraway, 1998), suggesting that effects of cattle grazing on small mammals can be further transferred to other trophic levels through trophic cascade. Sometimes, small mammals also act as efficient ecological engineers and profoundly alter surrounding landscape (e.g. Davidson & Lightfoot, 2006). Considering the important roles that small mammals play in ecosystems, it is necessary to explore their response to grazing if we hope to fully understand the ecological consequences of cattle grazing and develop sustainable land use strategies.

Manipulated experiments have proved to be an important method to answer this question. Although there seems to be no simple answer, a large body of studies using cattle-proof enclosures or exclosures detects more or less negative effects of cattle grazing on small mammal species richness or diversity (e.g. Keesing, 1998; Eccard, Walther & Milton, 2000; Schmidt & Olsen, 2003; Giuliano & Homyack, 2004). However, most of these studies are based on relatively simple experimental design and only consider the presence/absence of grazing, thus unable to detect any complex relationship between small mammal communities and grazing intensity. From a practical viewpoint, an experimental design involving multiple levels of grazing intensity is also required if we want to find a suitable grazing intensity when developing sustainable rangeland use strategies.

In this study, we investigate small mammal communities under four grazing levels in the Hulunber meadow steppe. The Hulunber meadow steppe is one of the most important stockbreeding bases in China (Guo et al., 2010) and provides essential habitats for many wildlife (Wang et al., 1997). Just like in many other grassland systems, cattle grazing is one of the most commonplace types of land use in meadow steppe and can significantly alter plant biomass, habitat structure and plant species composition (e.g. Zhu et al., 2012; Yan et al., 2015). However, to this date, no study has yet assessed the response of small mammals to cattle grazing in this area, hindering a comprehensive understanding in the dynamics and functioning of the Hulunber meadow steppe. Our present study should be a starting point.

## Materials and Methods

### Study area

Our study area is located in a transitional zone between the foothills in western Greater Khingan Mountains and the Mongolian Plateau, with an altitude of 660–680 m (49°95′N, 119°33′E). The climate here is characterized by a long, severe winter and a wet, warm summer. Annual temperature is 2 °C, ranging from −25 °C in January to 19 °C in July. Average annual precipitation is about 250 mm, approximately 70% of which occurs in July, August and September. The soil substrate is Chernozem or chestnut soil and the vegetation is dominated by *Leymus chinensis*, *Stipa baicalensis*, *Carex pediformis*, *Galium verum*, and *Bupleurum scorzonerifolium*.

### Manipulation of grazing intensity

We manipulated grazing intensity by using twelve cattle-proof pens (each of 200 × 250 m^2^), 3 km away from the Xiertala farm in northeastern Inner Mongolia, China. These pens were owned and maintained by the Hulunber Grassland Ecosystem Observation and Research Station (Yan et al., 2015). Each pen received one of the four treatments: G0 (not grazed, 0.00 Animal unit/ha, an animal unit means 500 kg of cattle body weight, hereafter Au), G1 (lightly grazed, 0.23 Au /ha), G2 (intermediately grazed, 0.46 Au/ha) and G3 (heavily grazed, 0.92 Au/ha). Since 2009, pens of G1, G2 and G3 were grazed from late May to early October in each year while pens of G0 were kept inaccessible for cattle. Small mammals, however, as well as predators (e.g. *Vulpes vulpes*, *Mustela eversmanii*, *Bubo bubo* and *Buteo buteo*), were free to move through the pens.

### Data collection

We carried out five monthly trapping sessions in the pens from June to October, 2013. Each trapping session lasted for five consecutive days. To avoid the potential effects of moonlight on small mammal activity, live-trapping was conducted during new moon periods. Two transects spaced 100 m were placed in each pen and each transect consisted of 25 trapping stations set at 10 m interval. A locally-made wire cage (12 × 15 × 30 cm^3^) was placed at each station and baited with fried peanuts. A piece of cotton was placed in each cage to keep captured animals warm during the night. Traps were checked twice a day (900–1,000 h and 1,600–1,700 h) and each trapped animal was weighed, sexed, toe-clipped or marked with hair dye in a unique pattern (if captured for the first time), and then released at the point of capture immediately. No trap was left in the pens between sessions.

Habitat characteristics within each pen were measured within ten 1 × 1 m^2^ quadrates one day after each trapping session. The positions of the quadrates were close to ten trap stations randomly selected. We identified all the plant species, measured canopy height (to the nearest 1 cm) and estimated total plant coverage in each quadrate. We harvested all the aboveground vegetation in each quadrate and measured its dry weight (to the nearest 0.1 g) to get the aboveground plant biomass in each quadrate. More specifically, grass species were classified and their total aboveground biomass (determined by their total aboveground dry weight) was recorded. We further divided grass aboveground biomass by total aboveground plant biomass to obtain the proportion of grass in local plant community. We also measured the soil hardness in each quadrate by using a soil penetrometer (TYD-1; Hangzhou HR, Hangzhou, China). The soil hardness was represented by the force on unit area (in kg/cm^2^) required to insert the steel awl (length: 40 mm) of the penetrometer into the soil.

Our study adheres to the guidelines of the American Society of Mammalogists (Sikes et al., 2011). Ethics approval was given by the Animal Ethics Committees at the Institute of Zoology, Chinese Academy of Sciences. The approval number is IOZ14001.

### Statistical analysis

Our analyses were based on pen-level measures per session. For each session, the values of each habitat variable measured in each pen were pooled together and averaged. For each small mammal species, the number of different individuals captured in each pen per session was used as a surrogate of its monthly relative abundance. We used unique individuals instead of captures to avoid potential biases caused by difference in intrinsic recapture possibility among species. We did not use a population estimator (e.g. in Program MARK) here, since we were more interested in relative difference in abundance rather than estimations of absolute abundance. Species richness of small mammals was represented by bias-corrected Chao 2 index, an asymptotic richness estimator representing the lower end of potential species richness (Chao, 2005). Diversity of small mammals was expressed by Hill’s N_2_ or Simpson’s reciprocal index and Shannon’s diversity index (*H*′), with the former more sensitive to the abundance of abundant species and the latter more sensitive to the abundance of rare species. Hill’s N_2_ and Shannon’s diversity index were calculated as 1/*D* = 1/Σ*P*_*i*_^2^ (Hill, 1973) and *H*′ = −Σ*P_i_LnP_i_* (Shannon & Weaver, 1949), respectively.

To explore the effects of grazing level on small mammal communities, we conducted repeated measures ANOVA on abundance (sum of respective relative abundance for all the species captured, log transformed prior to ANOVA to meet the assumptions of ANOVA), Chao 2 richness index and Shannon’s diversity index (square root transformed), with grazing level as the between-subjects factor and month as the within-subjects factor. Tukey HSD tests were conducted for multiple comparisons. Similar ANOVA procedures were also used to test for the effects of grazing level for all the seven habitat variables (values of aboveground plant biomass and soil hardness were square root transformed prior to ANOVA). A significance level of 0.05 was adopted and mean values were reported as mean ± standard error throughout the paper.

In order to explore the role of habitat characteristics in shaping small mammal community structure, we conducted a canonical correspondence analysis (CCA) with species captured. CCA is a direct ordination method which relates variation in species assemblage (the dependent variables) to variation in habitat characteristics (the independent variables). CCA has been widely used in community ecology and its advantages and robustness have been verified (Palmer, 1993). In our study, the independent variables was a matrix with habitat variables (plant coverage, canopy height, plant species richness, aboveground plant biomass, aboveground grass biomass, grass proportion and soil hardness) as columns. The dependent variables included a matrix with abundance of small mammal species as columns. Since the influence of rare species on the analysis is often exaggerated in CA/CCA ordination, it is necessary to exclude rare species prior to CA/CCA (Legendre & Gallagher, 2001). One species (*Allactaga sibirica*) was therefore excluded from CCA procedure because of its too low capture rate.

We used EstimateS for Windows 9.1.0 (Colwell, 2013) to calculate the Chao 2 richness estimator. The other statistical work was performed by using the R statistical package (R Development Core Team, 2015) version 3.1.3 including the VEGAN package (Oksanen et al., 2016).

## Results

Table 1 summarizes habitat characteristics in pens with different grazing levels. Consistent with Yan et al. (2015), canopy height (F_3,6_ = 265.96, P < 0.001), coverage (F_3,6_ = 78.22, P = 0.001) and aboveground biomass (F_3,6_ = 170.56, P < 0.001) significantly decreased with increasing grazing intensity, while plant species richness was not significantly affected by grazing intensity (F_3,6_ = 2.28, P = 0.18). On the contrary, soil hardness monotonically increased with increasing grazing intensity (F_3,6_ = 48.68, P < 0.001, Table 1). In regards to the two variables related to grass, G0 pens were similar to G1 pens whilst G2 pens were similar to G3 pens (Table 1).

**Table 1:** A summary of habitat characteristics (mean value ± standard error) across four grazing levels (n = 15 for each) in the Hulunber meadow steppe. Means with different letters after the error data are statistically different (Tukey HSD tests for post-hoc comparisons).

Habitat variables	Grazing levels			
	G0(0.0 Au/ha)	G1(0.23 Au/ha)	G2(0.46 Au/ha)	G3(0.92 Au/ha)
Canopy height (cm)	21.18 ± 1.19a	18.65 ± 1.20a	10.52 ± 0.76b	9.11 ± 0.94b
Plant coverage (%)	59.76 ± 4.16a	59.94 ± 3.03a	50.54 ± 2.65ab	47.70 ± 2.34b
Aboveground plant biomass (g)	203.71 ± 12.15a	164.75 ± 11.39b	96.88 ± 5.58c	89.35 ± 10.15c
Plant species richness	16.22 ± 1.21a	17.07 ± 1.15a	18.28 ± 1.13a	18.40 ± 1.31a
Aboveground grass biomass (g)	71.95 ± 13.20a	64.77 ± 10.38a	19.37 ± 1.99b	15.93 ± 1.84b
Grass proportion (%)	33.04 ± 5.36	37.13 ± 4.49a	20.04 ± 1.88	19.27 ± 1.96
Soil hardness (kg/cm^2^)	265.99 ± 7.25c	313.25 ± 6.92b	361.85 ± 11.14a	396.03 ± 13.68a

In total, we conducted 15,000 trapping days and 326 individuals representing five small mammal species were captured: *Spermophilus dauricus* (daurian ground squirrel, 189 individuals), *Ochotona dauurica* (daurian pika, 55 individuals), *Microtus gregalis* (narrow-headed vole, 43 individuals), *Cricetulus barabensis* (striped hamster, 33 individuals) and *Allactaga sibirica* (five-toed jerboa, 6 individuals). No individual appeared in more than one pen. There were 34 *S. dauricus* and 7 *M. gregalis* captured in more than one session.

**Figure 1:**
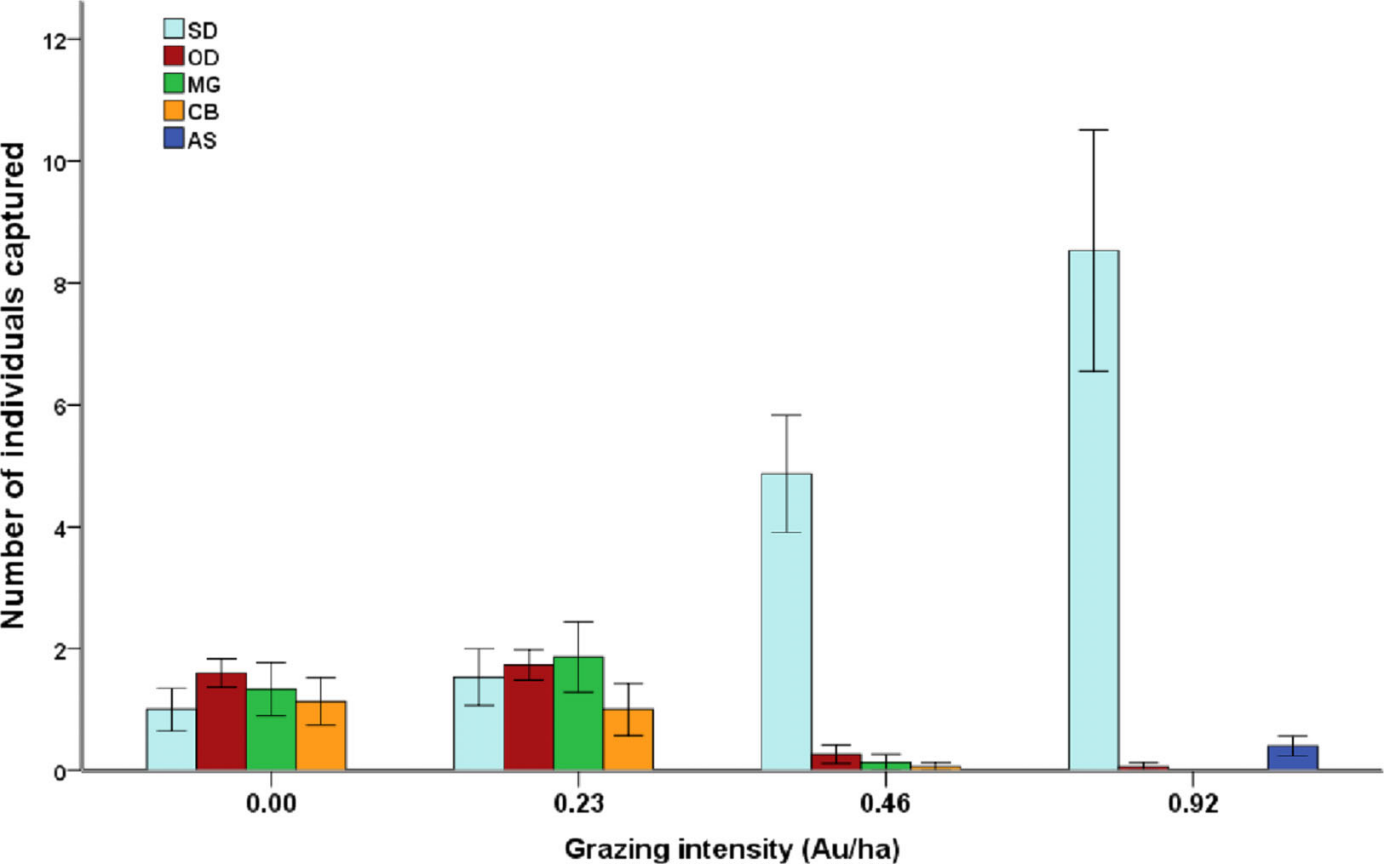
Species composition of small mammal communities across four grazing levels. Mean value ± standard error of relative abundance (represented by number of individuals captured) are presented by different species (SD, *Spermophilus dauricus*; OD, *Ochotona dauurica*; MG, *Microtus gregalis*; CB, *Cricetulus barabensis*; AS, *Allactaga sibirica*).

Cattle grazing dramatically affected local small mammal communities (Fig. 1; Table 2). *A. sibirica* was found exclusively in G3 pens. *S. dauricus* was significantly more frequently captured in G2 and G3 pens than in G0 and G1 pens, while *O. dauurica*, *C. barabensis* and *M. gregalis* showed the opposite pattern (Fig. 1; Table 2). Species richness (Chao 2 index: F_3,6_ = 39.07, P < 0.01) and diversity (Shannon’s index: F_3,6_ = 23.27, P < 0.01; Hill’s N_2_: F_3,6_ = 24.58, P < 0.01) varied among grazing levels, with all the indices significantly higher in G0 and G1 pens than in G2 and G3 pens (Table 2). However, no significant relationship was detected between total abundance of small mammals and grazing level (F_3,6_ = 2.58, P = 0.15, Table 2).

**Table 2:** A summary of small mammal community structure (mean value ± standard error) across four grazing levels (n = 15 for each) in the Hulunber meadow steppe. Relative abundance was represented by number of individuals captured in a pen. Means with different letters after the error data are statistically different (Tukey HSD tests for post-hoc comparisons).

Community parameters	Grazing levels			
	G0(0.0 Au/ha)	G1(0.23 Au/ha)	G2(0.46 Au/ha)	G3(0.92 Au/ha)
Abundance	5.07 ± 0.76a	6.13 ± 0.92a	5.33 ± 1.03a	9.00 ± 2.09a
Chao 2 richness index	2.86 ± 0.30a	3.05 ± 0.18a	1.82 ± 0.34b	1.52 ± 0.19b
Shannon’s diversity index (*H′*)	0.68 ± 0.13a	0.79 ± 0.08a	0.12 ± 0.07b	0.11 ± 0.04b
Hill’s N_2_ index	2.09 ± 0.23a	2.13 ± 0.14a	1.13 ± 0.08b	1.07 ± 0.03b
*S. dauricus* (relative abundance)	1.00 ± 0.35b	1.53 ± 0.47b	4.87 ± 0.96a	8.53 ± 1.98a
*O. dauurica* (relative abundance)	1.60 ± 0.24a	1.73 ± 0.25a	0.27 ± 0.15b	0.07 ± 0.07b
*M. gregalis* (relative abundance)	1.33 ± 0.43a	1.87 ± 0.58a	0.13 ± 0.13b	0.06 ± 0.00b
*C. barabensis* (relative abundance)	1.13 ± 0.39a	1.00 ± 0.43a	0.07 ± 0.07b	0.00 ± 0.00b

**Figure 2:**
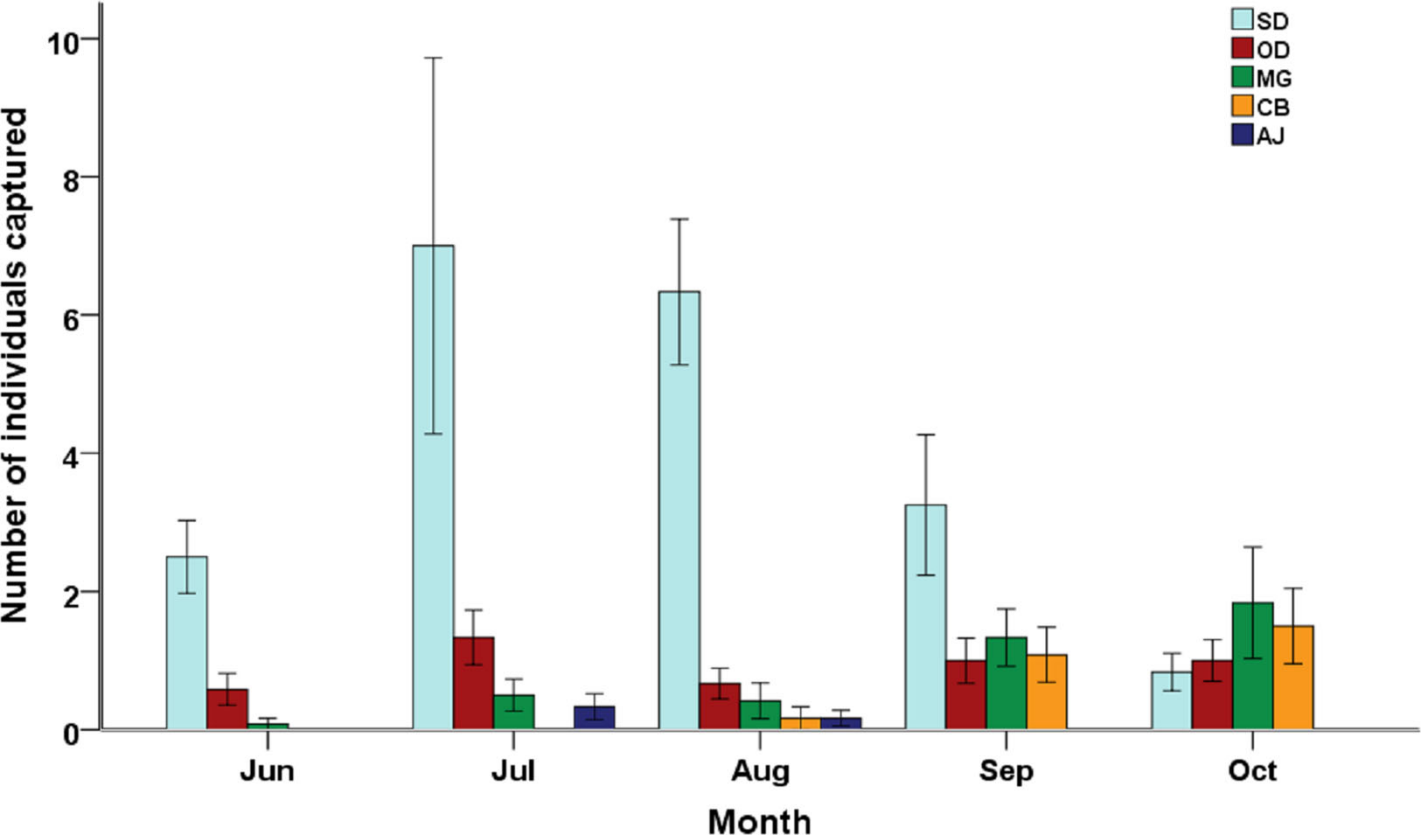
Species composition of small mammal communities across five months. Mean value ± standard error of relative abundance (represented by number of individuals captured) are presented by different species (SD, *Spermophilus dauricus*; OD, *Ochotona dauurica*; MG, *Microtus gregalis*; CB, *Cricetulus barabensis*).

Temporal variation in small mammal community also existed (Fig. 2). Small mammals were least abundant in June (38 individuals captured) but soon reached their peak in number in July (110 individuals captured). *S. dauricus* was most abundant in July (84 individuals captured) and least abundant in October (10 individuals captured), while *M. gregalis* and *C. barabensis* were most abundant in October (22 and 18 individuals captured, respectively) and least abundant in June (one and zero individual captured, respectively). Significant differences among months were detected in small mammal abundance (F_4,32_ = 10.64, P < 0.001), species richness (Chao 2 index: F_4,32_ = 17.10, P < 0.001) and diversity (Shannon’s index: F_4,32_ = 5.03, P < 0.01; Hill’s N_2_: F_4,32_ = 6.66, P < 0.01). On average, small mammal diversity and species richness reached their maximum values (Shannon’s index: 0.65 ± 0.15; Hill’s N_2_: 2.04 ± 0.27; Chao 2 index: 3.01 ± 0.44, respectively) in September and minimum values in June (Shannon’s index: 0.16 ± 0.08; Hill’s N_2_: 1.19 ± 0.10; Chao 2 index: 1.40 ± 0.22, respectively).

**Figure 3:**
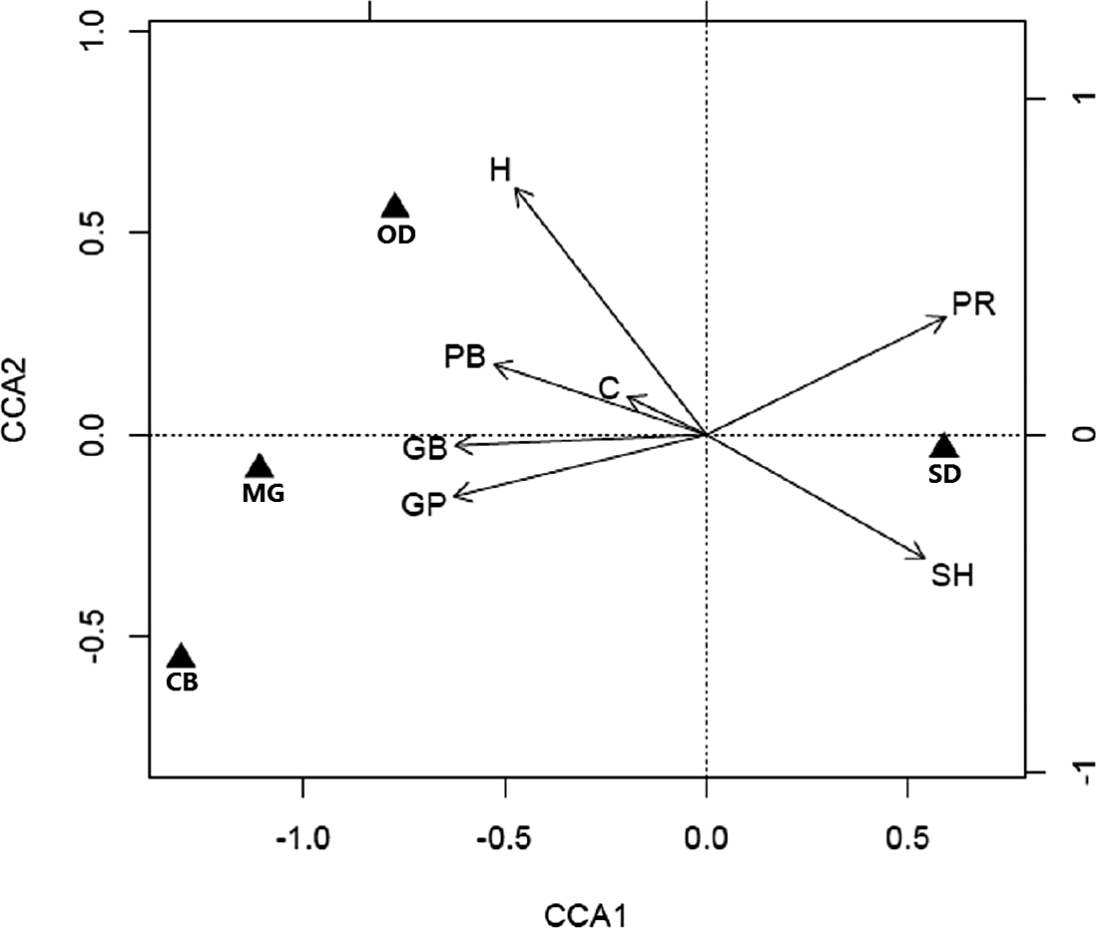
Biplots of canonical correspondence analysis (CCA) linking habitat characteristics with small mammal abundance. Abundance of *Allactaga sibirica* is not included in this analysis because of its too low trapping frequency. Triangles represent the optimal niches of four species (SD, *Spermophilus dauricus*; OD, *Ochotona dauurica*; MG, *Microtus gregalis*; CB, *Cricetulus barabensis*) in regard to the first and second CCA axes. Habitat variables are represented by vectors (H, canopy height; C, plant coverage; PB, aboveground plant biomass; PR, plant species richness; GB, aboveground grass biomass; GP, grass proportion; SH, soil hardness). The strength of the habitat-species relationship is indicated by distance between triangles (species) and vectors (habitat characteristics).

The CCA based on 1,000 Monte Carlo permutations suggested that a significant relationship between habitat variables and species abundance existed for the first canonical axis (CCA axis 1: eigenvalue = 0.62, P = 0.001; Fig. 3). This canonical axis alone explained 53% of total variance in small mammal abundance data. CCA axis 1 was positively related to plant species richness (R = 0.71) and soil hardness (R = 0.65), and negatively related to canopy height (R = −0.57), plant coverage (R = −0.23), aboveground plant biomass (R = −0.63), aboveground grass biomass (R = −0.75) and grass proportion (R = −0.75). We found that all of the four species possessed CCA scores larger than 0.5 on this axis (*S. dauricus*: 0.59; *O. dauurica*: −0.77; *M. gregalis*: −1.11 and *C. barabensis*: −1.30). The abundance of *S. dauricus* was positively related to soil hardness and plant species richness, while negatively related to other habitat variables. On the contrary, the abundance of *O. dauurica* was positively related to canopy height, plant coverage and aboveground plant biomass, while the abundance of *M. gregalis* and *C. barabensis* were most closely associated with grass proportion (Fig. 3).

## Discussion

Our data indicate that four-year manipulation of cattle grazing in the Hulunber meadow steppe resulted in dramatic changes in both habitat characteristics and the small mammal community. Generally, as grazing intensity increases to 0.46 Au/ha, grass dramatically decreases both in quantity and in proportion. Such alteration in vegetation structure, may have profound impacts on small mammal communities since structural complexity provided by vegetation is often crucial for small mammals (Kerley, 1992; Fitzherbert et al., 2007; Avenant & Cavallini, 2007). Our results clearly support this: when grazing intensity reaches 0.46 Au/ha, small mammal diversity significantly decreases, and *S. dauricus* becomes dominant in number. Consistent with many previous studies (e.g. Bakker, Olff & Gleichman, 2009; Bueno et al., 2012), response of small mammals to grazing is variable among species. *S. dauricus* and *A. sibirica* seem to prefer habitats with lower plant coverage and canopy height, contrary to the other three species. According to trapping records, *S. dauricus* and *O. dauurica* are two most common species widely distributed in the study area (both species appear in all treatments). However, *S. dauricus* and *O. dauurica* show quite different habitat association, with the former preferring heavily grazed grassland and the latter preferring intact or lightly grazed grassland. Both species are abundant in this region and their densities are easy to estimate, which makes them suitable indicators of ecological integrity. Using small mammals as ecological indicators is both effective and easy (Avenant & Cavallini, 2007) and it is therefore valuable to keep monitoring population dynamics of these species.

We think that perceived predation risk may play an important role in determining these different patterns of habitat selection. Previous studies suggest that predation risk often accounts for a large portion of foraging costs perceived by small mammals (e.g. Brown, Kotler & Valone, 1994) and the pattern of habitat/microhabitat use in many small mammals can be partly explained by predation risk (e.g. Grant et al., 1982; Cassini & Galanthe, 1992; Eccard, Walther & Milton, 2000). Some of the difference in predation risk perceived by small mammals can be related to the difference in morphology, locomotion modes and anti-predation strategies (Kotler, Brown & Mitchell, 1994). Larger body size is often related to faster movement and bipedal locomotion allows for changing directions more erratically while escaping from predators (Djawdan & Garland, 1988). These two characteristics therefore might be favored in the open microhabitat but at the cost of lower foraging efficiency in bush microhabitat, which is safer for small-size and quadrupedal species (Brown et al., 1988). We think, this may also be the case in our system. Being a large-sized bipedal species, *A. sibirica* is morphologically similar to kangaroo rats in North America and it is not surprising to find it exclusively on open area such as the heavily grazed pens in our study. *S. dauricus* is the largest rodent species in the study area. Like other ground squirrels, it often stands on its hindlegs to watch around when vigilant (Ling-Ying Shuai, 2011–2013, personal observation). This suggests that, *S. dauricus* may rely on early detection of predators and fast speed rather than vegetation cover to decrease predation risk. Tall grass or thick vegetation cover may not be very effective in providing refuges for large species such as *S. dauricus* but instead prevent it from successfully detecting approaching predators, especially terrestrial carnivores. Thick vegetation cover may also decrease running speed of ground squirrels and further increase their perceived risk (Schooley, Sharpe & Van Horne, 1996). On the contrary, voles (e.g. *M. arvalis* and *M. agrestis*) are well-known to prefer thick herbaceous cover and rely on shortcuts under cover to quickly retreat to their burrows (e.g. Jacob & Brown, 2000; Fey, Banks & Korpimäki, 2006). As a result, changes in habitat structure caused by overgrazing may generate small mammal communities dominated by large-sized species, whose perceived risk is low in heavily grazed area.

Other ecological factors, such as interspecific competition, may also be important in shaping small mammal community. In this sense, the scarcity of species such as *M. gregalis* and *O. dauurica* in highly grazed area may be attributed to the competitive exclusion by *S. dauricus*, the individually dominant species. This seems reasonable since our trapping data suggests the existence of temporal partitioning among these species on a seasonal basis, with different species reaching its peak in different season. If this is true, the number of *M. gregalis* and *O. dauurica* should increase in the heavily grazed area, once *S. dauricus* is removed. Although we have not conducted experiments to directly test this prediction, we think it is less likely to happen. *M. gregalis* and *O. dauurica* are either small in body size or quadrupeal (which is also true for *C. barabensis*), which makes them less efficient in detecting and escaping from predators when foraging in open microhabitat. In this sense, overgrazed grassland is still not a suitable habitat for them even if *S. dauricus* is absent. However, one of our previous experiments (Ling-Ying Shuai, 2011–2013, personal observation) suggests that interference competition does exist between *S. dauricus* and *M. gregalis*, with the latter dramatically decreasing its activity level when the former is present. It seems that in our system, interspecific competition plays an important role in temporal partitioning rather than in habitat selection.

Our experimental design involves multiple levels of disturbance (four levels of grazing intensity) and thus enables us to assess the validity of the intermediate disturbance hypothesis (the IDH), a classic ecological theory but associated with long-term debates (Mackey & Currie, 2001). The IDH predicts that communities under intermediate intensities or frequencies of disturbance should possess higher species diversity than that under low levels of disturbance or severe disturbance (Connell, 1978). In the present study, cattle grazing can be viewed as a type of chronic and regular disturbance. If the IDH is correct, species richness or diversity in native small mammal community should be significantly higher under light or intermediate grazing pressure than under two extremes of grazing intensity (not grazed and heavily grazed). Our study does not fully support the IDH. Although, G1 and G2 treatments possess significantly higher small mammal species richness and diversity than G3 treatment, none of these two treatments produce significantly higher small mammal species richness or diversity than G0 treatment. In other words, the peaked pattern predicted by the IDH does not actually appear.

The alteration in small mammal communities associated with cattle grazing may bring profound effects to the other parts of local ecosystems. For example, *S. dauricus* is active only during daytime (Shuai et al., 2014) while *C. barabensis* is mainly nocturnal and *M. gregalis* and *O. dauurica* are active in both daytime and nighttime (Ren, 2010). A community dominated by *S. dauricus* may therefore be beneficial for diurnal predators such as *B. buteo* but disadvantageous for nocturnal and crepuscular predators such as *B. bubo* and *V. vulpes*. In this sense, alteration in small mammal community structure may cause further changes in local fauna through trophic cascade. Although, we have not conducted experiments to directly assess the response of predators, we think such effects are likely to happen, considering that activity patterns are often evolutionarily constrained (Kronfeld-Schor et al., 2001; Roll, Dayan & Kronfeld-Schor, 2006), and predators well adapted for nocturnal hunting are often less adapted to forage during daytime (Kronfeld-Schor & Dayan, 2003). On the other hand, the changes in small mammal community may be further mediated to plant community and landscape structure. Previous studies find that available burrow densities of plateau pika (*O. curzoniae*) significantly affect characteristics and distribution pattern of plant, soil organic carbon and nitrogen content in *Kobresia pygmaea* community (Pang et al., 2015a; Pang et al., 2015b). Net ecosystem carbon exchange and soil moisture in alpine meadow steppe are also significantly affected by population density of *O. curzoniae* (Liu et al., 2013). In our system, *O. dauurica* is similar to *O. curzoniae* both in ecology and morphology, and can be expected to play similar ecological roles. The alteration in abundance and distribution of *O. dauurica* caused by cattle grazing should also have profound implications in some aspects of the Hulunber meadow steppe. Further studies are required to explore these possible outcomes.

Practically, anthropogenic disturbance is often unavoidable and a critical step is thus to find a suitable frequency and intensity of disturbance. In our study, small mammal communities under light grazing intensity (0.23 Au/ha in the present study) possess highest diversity. This grazing level also helps to maintain the aboveground grass biomass and grass proportion. However, when grazing intensity increases to only 0.46 Au/ha, things greatly change. In terms of both the plant and the small mammal community, a critical transition point seems to exist between two disturbance levels (0.23 Au/ha and 0.46 Au/ha, Table 1; Fig. 1). Meanwhile, changes in grass proportion caused by cattle grazing seem to be important in shaping small mammal communities. These results should provide useful information for developing suitable land use strategies, given that small mammals play an important role in shaping the structure and functioning of ecosystems.

## Supplemental Information

10.7717/peerj.2349/supp-1 Supplemental Information 1 A summary for habitat characteristics and small mammal communities.

## Additional Information and Declarations

### Data Deposition

The following information was supplied regarding data availability: The raw data has been supplied as Supplemental Dataset Files.
